# Measurement of alienation among adolescents: construct validity of three scales on powerlessness, meaninglessness and social isolation

**DOI:** 10.1186/s41687-018-0040-y

**Published:** 2018-03-16

**Authors:** Signe Boe Rayce, Svend Kreiner, Mogens Trab Damsgaard, Tine Nielsen, Bjørn Evald Holstein

**Affiliations:** 1 VIVE – the Danish Center for Social Science Research, Herluf Trolles Gade 11, 1052 Copenhagen K, Denmark; 2 Department of Biostatistics, Institute of Public Health, University of Copenhagen, Øster Farimagsgade 5, P.O.B. 2099, 1014 Copenhagen K, Denmark; 3 National Institute of Public Health, University of Southern Denmark, Studiestræde 6, 1455 Copenhagen K, Denmark; 4 Department of Psychology, University of Copenhagen, Øster Farimagsgade 2A, 1353 Copenhagen K, Denmark

**Keywords:** Adolescents, Alienation, Construct validity, Item response theory, Meaninglessness, Powerlessness, Rasch models, Social isolation, Scale validation

## Abstract

**Background:**

Psychological alienation is an important concept in the study of adolescents’ health and behavior but no gold standard for measuring alienation among adolescents exists. There is a need for new scales with high validity for use in adolescent health and social research. The purpose of the present study was to develop and validate alienation scales in accordance with Seeman’s conceptualization of alienation focusing on three independent variants specifically relevant in adolescent health research: powerlessness, meaninglessness and social isolation.

**Methods:**

Cross-sectional data from 3083 adolescents aged 13 to 15 years from the Danish contribution to the cross-national study Health Behaviour in School-aged Children (HBSC) were used. We identified and developed items, addressed content and face validity through interviews, and examined the criterion-related construct validity of the scales using graphical loglinear Rasch models (GLLRM).

**Results:**

The three scales each comprised three to five face valid items. The powerlessness scale reflected the adolescent’s expectancy as to whether his/her behavior can determine the outcome or reinforcement he/she seeks. The meaninglessness scale reflected the expectancy as to whether satisfactory predictions regarding the effects of one’s behavior are possible. Finally, the social isolation scale reflected whether the adolescent had a low expectancy for inclusion and social acceptance. All scales contained some uniform local dependency and differential item functioning. However, only to a limited degree, which could be accounted for using GLLRM. Thus the scales fitted GLLRMs and can therefore be considered to be essentially construct valid and essentially objective.

**Conclusion:**

The three alienation scales appear to be content and face valid and fulfill the psychometric properties of a good construct valid reflective scale. This suggests that the scales may be appropriate in future large-scale surveys to examine the relation between alienation and a range of adolescent health outcomes such as health, behavior and wellbeing.

**Electronic supplementary material:**

The online version of this article (10.1186/s41687-018-0040-y) contains supplementary material, which is available to authorized users.

## Background

Alienation among adolescents has been described as ‘*…to lack a sense of belonging, to feel cut off from family, friends, school or work – the four worlds of childhood*’ by Bronfenbrenner [1] and is a serious psychological strain for a young person. Supportive social relations and empowerment are of extraordinary importance for positive youth development [2] and the negative psychosocial consequences of social withdrawal and rejection during adolescence have been emphasized in the literature [3–6]. Some studies indicate that the affective consequences are greater among adolescents compared with adults [7].

Alienation is an important concept in the study of adolescent behavior and health because the feelings of alienation may reflect a living situation, which harms a healthy development during adolescence. It is therefore important with sound scales for the measurement of alienation among adolescents.

Alienation has been treated as both a psychological and a sociological concept. The sociological concept focuses on the social *processes* that lead to alienation while the psychological concept focuses on the subjective *feelings* of alienation. These feelings may be caused by social processes but not necessarily so [8]. There is no universal definition of alienation. Because ‘alienation’ denotes different ideas, and uncertainties about the meaning of the word and concept easily arise, it has been described as an ambiguous concept [8]. Most research on alienation as a psychological concept builds upon Seeman’s conceptualization [9–11]. Seeman identified and described variants of alienation based on the understandings in which alienation had been used theoretically and empirically. The aim was to clarify the variants of alienation and thereby facilitate the use of the concept [8, 10]. The conceptualization comprises six alternative variants of alienation: powerlessness, normlessness, meaninglessness, self-estrangement, cultural estrangement and social isolation [8, 10]. All six variants are aspects of the individual’s personal expectations and values. According to Seeman alienation should be studied by addressing the specific variants and not as a joint measure [12]. As the six alienation variants are alternative *variants*, there is no demand of combining the six variants to study alienation. Likewise, there is no theoretical structure between the alienation variants. Some variants can, be related to each other [8, 10].

Alienation is predictive of deviant behavior such as truancy, bullying, drug use, crime and suicide [9, 13–17]. It is also predictive of psychological and health-related outcomes and behavior such as physical and psychological symptom load, life satisfaction, alcohol use, drunkenness, less exercising and eating unhealthy food [17–21].

While relevant in health-related research, the measurement of alienation is challenging. Alienation has been measured in various ways, but no gold standard exists. Several scales which build on Seeman’s conceptualization have been applied in adolescent research [20, 22–26]. Despite their common conceptual background, there is considerable heterogeneity in measurement and how the scales were validated. Previous scales differ in variants of alienation included. They also differ in whether alienation is operationalized as a single scale of general alienation [20, 23], as separate scales for each alienation variant [24] or by combining subscales into one joint alienation scale [22, 25, 26]. Likewise, there are differences in how the scales are validated statistically ranging from information about reliabilities and convergent validity only [22] to investigation of construct validity using confirmatory factor analysis [26, 27], comparison of the pattern of group difference to the pattern expected from theory [24] or by testing for differential item functioning [20].

However, there is still a need for development of new scales with high validity for use in adolescent health and social research. It is important to avoid systematic bias in measurement by ensuring criterion-related construct validity. According to modern item response theory (IRT), a scale should meet four validity criteria: unidimensionality, monotonicity, local independence between the items of the scale, and no differential item functioning (DIF) in relation to other variables than those include in the scale [28]. Despite the first demand of a scale being unidimensional, alienation has mainly been measured either by multidimensional scales or by combining subscales into a multidimensional scale [20, 22, 23, 25, 26]. We have not been able to identify any alienation scales tested according to these more demanding criteria of modern item response theory.

This study contributes to the field by developing new scales in accordance with Seeman’s conceptualization focusing on three independent variants of alienation specifically relevant in adolescent health research: the feelings of powerlessness, meaninglessness and social isolation. We examine the construct validity of these three scales using modern item response theory: graphical loglinear Rasch models (GLLRM).

## Methods

The data come from the Danish contribution to the international Health Behaviour in School-aged Children (HBSC) study 2010 [29], an international cross-sectional WHO collaborative study. The overall purpose was to increase understanding of young people’s health and lifestyle in their social context [30].

### Sample

The Danish HBSC study 2010 comprised pupils from the fifth (mean age 11.8 years, SD = 0.44), seventh (13.8 years, SD = 0.48) and ninth grade (15.8 years, SD = 0.42) in 75 randomly selected schools. The pupils completed the internationally standardized HBSC questionnaire [31, 32] in school. Participation rate was 98.7% of the pupils present and 86.3% of the pupils formally enrolled in the target classes (*n* = 4922). The present study included data from seventh and ninth grade pupils (*n* = 3083, 1541 girls and 1542 boys).

There is no formal agency for ethical approval of school surveys in Denmark. Therefore we asked the school leader, the board of pupils, and the board of parents in each of the participating schools for assessment and approval of the study. The pupils received oral and written information about the study and that participation was voluntary and anonymous.

### Measurements

#### Alienation

Feeling empowered and socially related are of extraordinary importance for a healthy psychosocial development in adolescence [2–6]. Three of Seeman’s alienation variants [10, 11] are specifically relevant in an adolescent setting since feeling powerlessness, meaninglessness and socially isolated may pose a threat to adolescent psychosocial health. For this reason, we focused on developing scales for these three alienation variants.

The development of the three scales was an iterative process starting with a thoroughly conducted conceptual work. This included a review of the literature on alienation and related concepts in order to reach a clear understanding and conceptualization of adolescent alienation. While no universal definition of alienation exists, most measurements and studies on adolescent alienation were founded in Seeman’s conceptualization of psychological alienation, or addressed similar experiences.

The choice of items was based on several considerations. Conceptually the items had to reflect central aspects of Seeman’s alienation variants: powerlessness, meaninglessness and social isolation. We searched for and compiled a pool of items which appeared to have a conceptual potential to measure these three alienation variants. The content and appropriateness of these items was discussed within the research group, which has expertise in adolescent psychosocial health. Based on these discussions, adaptions of the items were made in order to fit the context and the age group. The first version of the items was tested through pilot tests where pupils answered the questionnaire and subsequently participated in qualitative focus group interviews. A moderator and an observer from the HBSC research team, including the first author, participated in these interviews. The interviews were conducted separately for each class level and divided into three groups (boys only, girls only and a mixed group).Six interviews were conducted among the 13- and 15-year-olds comprising 44 adolescents (13-year olds: 21, 15-year-olds: 23). We extracted content about item relevance and face validity. Further, initial psychometric analyses were conducted based on the larger pilot sample in order to identify potentially unrelated items. We excluded items which seemed irrelevant to the pupils, had low face validity or did not seem to belong to the chosen alienation variants based on the psychometric analyses. We chose to exclude the youngest age group (11-year-olds) because the content of some items was either difficult to understand or irrelevant to them. Finally, the scales were pilot tested in a seventh, eighth and ninth grade (13-, 14- and 15-year-olds) before they were used in the HBSC-study 2010.

We measured powerlessness, meaninglessness and social isolation by three reflective scales, i.e. scales where the included items reflect the concept measured and where the responses on the items are causally dependent on the latent variable (here: the alienation variant) [33]. Each scale comprised three to five trichotomized items reflecting the respective alienation variant in question. Based on conceptual considerations, we trichotomized the response categories into high, medium and low degree of alienation aiming to create a conceptual symmetry in the responses. ‘High’ reflected response categories which the authors assessed as indicators of alienation, ‘low’ reflected that the responses did not indicate alienation, and ‘medium’ denoted the remaining response categories.

The alienation variant *powerlessness* is characterized by ‘the expectancy or probability held by the individual that his own behavior cannot determine the occurrence of the outcomes, or reinforcements, he seeks’ [10]. The powerlessness scale included three items reflecting such expectancies: ‘Cannot overcome problems’, ‘Cannot manage things’ and ‘Feeling helpless’.

Seeman defined the alienation variant *meaninglessness* as ‘a low expectancy that satisfactory predictions about future outcomes of behavior can be made’ [10] i.e. whether it is possible to make satisfactory predictions regarding the effects of one’s behavior. Regarding adolescents, Mau noted that meaninglessness indicate a lack of connectedness between the present and the future, e.g. by perceiving a limited relationship between academic performance and a future job [12]. Meaningless was measured by a scale comprising the three items: ‘Things have no meaning’, ‘Not knowing what is going on’ and ‘School is not preparing me for the future’.

The alienation variant *social isolation* refers to a low expectancy for inclusion and social acceptance [11] A consequence of this can be loneliness and a feeling of being rejected [11]. Social isolation was measured by a five-item scale reflecting these feelings. The five items were: ‘Feeling left out’, ‘Not feeling close to family’, ‘No support when unhappy’, ‘Feeling lonely’ and ‘Not belonging’.

Table 1 shows the exact phrasing of the items, the trichotomization of the response categories, and source of inspiration.

**Table 1:** Phrasing and trichotomization of variables translated into English

Alienation variant	Variable name	Phrasing	Response categories		
			High	Medium	Low
Powerlessness	Cannot overcome problems^a^	If you try hard enough, how often do you know what to do to overcome a problem?	‘Never’ and ‘Rarely’ (know what to do)	‘Sometimes’	‘Often’ and ‘Always’
	Cannot manage things^a^	How often can you manage things you set out to do?	‘Never’ and ‘Rarely’ (able to manage things)	‘Sometimes’	‘Often’ and ‘Always’
	Feeling helpless^a^	How often do you feel helpless?	‘Always’ and ‘Often’	‘Sometimes’	‘Rarely’ and ‘Never’
Meaninglessness	Things have no meaning^a^	How often do you feel there is little meaning in the things you do in your daily life?	’Very often’ and ‘Often’	‘Sometimes’	‘Seldom’ and ‘Never’
	Not knowing what is going on^a^	How often do you feel you don’t really know what is happening?	’Very often’ and ‘Often’	‘Sometimes’	‘Seldom’ and ‘Never’
	School is not preparing me for the future^b^	School prepares me for what I want to do when I leave.	‘Strongly disagree’ and ‘Disagree’ (that school prepares me)	’Neither agree nor disagree’	’Agree’ and ‘Strongly agree’
Social isolation	Feeling left out^a^	How often do you feel left out of things?	‘Always’ and ‘Often’	‘Sometimes’	‘Rarely’ and ‘Never’
	Not feeling close to family^b^	Do you feel close to your family?	‘Never’ and ‘Rarely’	‘Sometimes’	‘Always’ and ‘Often’
	No support when unhappy^b^	Are there people you can turn to for support when you are unhappy?	‘Never’ and ‘Rarely’	‘Sometimes’	‘Often’ and ‘Always’
	Feeling lonely^a^	Do you ever feel lonely?	‘Yes, very often’ and ‘Yes, often’	’Yes, sometimes’	’No′
	Not belonging^b^	I feel I belong to several different groups of friends.	‘Strongly disagree’ and ‘Disagree’	’Neither agree nor disagree’	’Agree’ and ‘Strongly agree’,

^a^Previous or adapted from previous HBSC questionnaires [30]

^b^Inspired by Mau [23]

#### Covariates

We included five covariates in the analyses. Physical inactivity as we intend to study physical inactivity in relation to alienation, and four potential confounders of this association: sex, age group, socio-economic position (SEP) and migration status [34, 35].

We measured physical inactivity by combining two items on vigorous physical activity (VPA) and moderate to vigorous physical activity (MVPA), respectively. These two items have been found valid and reliable in previous studies [36, 37]. Physical inactivity was defined as 0–½ hour of VPA a week combined with 0–1 day a week with 60+ minutes of MVPA. Pupils with missing VPA and/or MVPA were coded missing unless VPA or MVPA gave evidence of physical activity.

Age group was measured by grade as the pupils were age-homogeneous within grades. SEP was measured by pupil-reported parental occupation and coded according to the standards of the Danish National Institute of Social Research into occupational social class (OSC) I (highest) to V, group VI (employed but insufficient information for the coding of OSC) and VII (economically inactive). We categorized the pupils by the highest-ranking parent. Migration status was measured by the pupil’s and parents’ country of birth and coded into: 1) Danish origin, 2) descendants of immigrants and 3) immigrants.

### Scale development

In recent decades a growing number of scale validity studies have employed modern test theory such as item response theory (IRT) focusing on the issues of item fit, DIF and requirements for measurement. Rasch models (RM) is a family of IRT models for dichotomous and polytomous items [38]. The Rasch family of models include the original RM for dichotomous items [39], the generalization of this into the polytomous RM [40], and the GLLRM [28].

In IRT four strict requirements must be met to ensure criterion-related construct validity of a scale to ensure there is no systematic bias in the measurement [28, 41]. Firstly, the scale must be *unidimensional* measuring one latent variable only. Secondly, there must be *monotonous relationship* between the latent variable score (theta) and the probability of item responses. Thirdly, items must be conditionally independent given the latent variable i.e. *local independence*. Last, there must be absence of *DIF* meaning that items and exogenous variables are conditionally independent given the latent score [42]; a person’s response on an item should depend on his or her level on the latent variable only and not on any other characteristic such as sex or age.

Rasch models are often used as gold standard expressing ideal measurement requirements because scales fitting an RM hold all the characteristics that follow from the above four requirements. Additionally, in scales that fit an RM the set of items are homogeneous meaning that the rank order of the item difficulties is the same for all respondents regardless of the level on the latent variable [43]; i.e. the most severe item is the most severe for everybody. Thus, items that fit an RM provide ideal measurement in the specific frame of reference (e.g. population). The measurement can be described as specifically objective, sufficient and therefore reliable [28, 33]. Specific objectivity refers to that comparisons of persons level on the latent variable do not depend systematically on which items of the scale we use, and that comparisons of items do not depend on which persons in the sample we use, within the specific frame of reference. Sufficiency means that the total score is sufficient, i.e. no additional information besides the total score can be gained from studying the response profile of the items [33]. The RM requires no assumptions about the latent variable being normally distributed as e.g. factor analysis models and furthermore tests the item fit of the individual items of the scale [33]. For all these reasons, we used RM to assess the psychometric properties of each of the four scales.

Most scales do present DIF and local dependency (LD). Testing such scales against an RM would result in exclusion of locally dependent items or items causing DIF even though these items appear to be face valid [28]. The consequence would be a reduced reliability of the scale. GLLRMs provide a solution to this loss of reliability. By adjusting for significant LD and DIF in the analyses face valid items can be kept in the scale. Thereby unnecessary loss of reliability can be avoided. Kreiner & Christensen [28] claim that in scales fitting GLLRMs the measurement is still essentially valid and essentially objective [28, 33].

### Statistical analyses

We tested the RM assumptions for each of the scales with the following test statistics: 1) conditional likelihood ratio (CLR) tests of response homogeneity to compare item parameter estimates among persons with low and high scores respectively [44], 2) CLR tests comparing item parameter estimates in sub-populations defined by exogenous covariates were used for global tests of DIF [44], 3) two types of tests for DIF of specific items relative to specific covariates were calculated: First, Mantel-Haenszel tests of conditional independence of the item and the covariate given the total score on all items and second, log linear CLR tests of the hypotheses that the item and the covariate are conditionally independent given the latent variable [45]; both procedures provide estimates of conditional odds-ratios describing the strength of the effect of the covariate on the item response, 4) Likewise, we tested the hypotheses of local independence among items given the rest score by Mantel-Haenszel tests and by loglinear CLR tests [45]. Finally, to test the overall fit of the item to the model we used 5) item fit statistics comparing the observed and expected correlations between an item and rest scores without the item [46].

When DIF and/or LD were found, the next step was to attempt to fit a GLLRM where uniform DIF and uniform LD is permitted. The adequacy of the GLLRM was tested in the same way as the adequacy of the RM.

Reliability is defined in two different ways: 1) as the ratio between the population variance of the true scores and the variance of the observed scores or, 2) as the correlations between tests and retests taken under the assumption that the underlying true scores have not changed and that the tests and retests are conditionally independent, given the true scores. True scores are by definition unobservable and for this reason it is not possible to set up experiments providing data that can be used to estimate reliability. Instead, classical test theory uses Cronbach’s Alpha which is known to provide a lower bound of the true reliability. In IRT where models describing the association between the latent variable and the true scores on one hand and item responses on the other hand are known, it is easy to set up Monte Carlo experiments where test-retest reliability can be estimated directly by using 10,000 simulated samples for the estimation. Harmon & Mesbah [47] describe such methods. We used these methods not only to estimate a simulated test-retest correlation, but also to estimate the correlation between true and observed scores since this correlation provides a better measure of the performance of the measurement instrument.

Only pupils who answered all items in a scale and all covariates were included in the scale validation analysis of the scale in question. The number of pupils included in the respective scale validation analysis were 2928 (powerlessness), 2879 (meaninglessness) and 2866 (social isolation).

### Statistical software

We used Digram software [48] for scale validation analyses and SAS 9.1 for the remaining analyses.

## Results

The sample comprised equally many boys and girls (girls: *n* = 1541, boys: *n* = 1542). The distribution of the covariates age group in the sample was 53.6% 13-year-olds and 46.4% 15-year-olds; 16.6% of the pupils came from a family with a low SEP (V and VII), 38.3% from a family with a middle SEP (III-IV), and 34.0% from a family with a high SEP (I -II). The majority of the sample was of Danish origin (89.4%); 10.6% were classified as immigrants or descendants; 10.6% of the pupils were classified as physically inactive.

Table 2 shows the percentages of pupils with medium or high degree of alienation for each item in the scales stratified by sex and age group.

**Table 2:** Percentage with high or medium indication of alienation item by item stratified by sex and age

Boys							Girls					
Alienation dimension and indicator of alienation	n		13-year olds (*n* = 824)		15-year olds (*n* = 718)		n		13-year olds (*n* = 828)		15-year olds (*n* = 713)	
			Degree of alienation		Degree of alienation				Degree of alienation		Degree of alienation	
	13-year olds	15-year olds	High	Medium	High	Medium	13-year olds	15-year olds	High	Medium	High	Medium
Powerlessness ^a^												
Cannot overcome problems	805	697	3.4	15.9	3.0	10.6	816	702	5.6	17.7	2.6	13.5
Cannot manage things	804	697	2.1	17.6	2.3	12.2	813	702	2.7	20.7	2.4	17.4
Feeling helpless	800	696	3.8	8.4	4.0	9.8	813	702	5.2	16.1	3.7	21.4
Meaninglessness ^a^												
Things have no meaning	791	691	18.2	35.9	23.9	36.6	807	691	18.1	36.3	17.2	40.2
Not knowing what is going on	791	690	11.9	32.5	15.7	32.2	813	692	16.0	31.0	13.0	37.0
School is not preparing me for the future	783	690	13.2	21.5	13.0	23.0	807	688	13.1	26.0	9.9	24.3
Social isolation ^a^												
Feeling left out	803	699	3.7	10.5	6.0	10.9	815	700	4.9	19.5	3.6	18.7
Not feeling close to family	804	697	2.9	7.7	4.5	10.3	812	699	4.9	8.9	4.3	13.0
No support when unhappy	802	694	4.6	7.7	5.0	7.2	812	699	3.2	6.0	3.0	5.6
Feeling lonely	818	713	3.9	19.3	4.6	21.0	823	709	6.6	30.1	5.1	29.8
Not belonging	789	691	10.9	16.7	11.4	18.7	809	689	13.0	22.4	11.9	20.9

^a^Not all 3083 pupils had complete item information. Only pupils who answered all items in the scale and covariates were included in the scale validation analysis in question

Generally, the prevalence and degree of alienation varies considerably among items within the scales, e.g. in the meaninglessness scale 60.5% (high: 23.9%; medium: 36.6%) of the 15-year-old boys experience a high or medium degree of alienation in relation to the item ‘Things have no meaning’ while 36.0% (high: 13.0%; medium: 23.0%) experience alienation in relation to the item ‘School is not preparing me for the future’. In relation to the social isolation scale overall 32.8% (high: 11.9%; medium: 20.9%) of the 15-year old girls experience alienation in relation to the item ‘Not belonging’ while the corresponding prevalence is only 8.6% (high: 3.0%; medium: 5.6%) in relation to the item ‘No support when unhappy’.

In relation to sex, the prevalence of alienation within the specific scales differed between boys and girls as follows: *Powerlessness:* A significantly larger percentage of the girls ‘feel helpless’ (*p* < 0.001) and ‘cannot manage things’ (*p* < 0.001) in a medium degree. *Meaninglessness:* The prevalence of high and medium degrees of alienation is generally high among both sexes. There is a tendency toward more boys than girls feel that ‘Things have no meaning’ in a high degree, however, this is only significant at a 5% level of significance (*p* = 0.029). *Social isolation:* A significantly larger percentage of girls experience a medium degree of alienation for the items ‘Feeling left out’ (*p* < 0.001), ‘Feeling lonely’ (*p* < 0.001) and ‘Not belonging’ (*p* = 0.01). There is a tendency toward a higher degree of alienation among girls for most items. For the item ‘No support when unhappy’ significantly more boys than girls feel alienated in a high degree on a 5% level of significance (*p* = 0.017).

### Local dependency and DIF

The test of fit of the RM showed significant LD and DIF in the final models for all three scales. These results are shown in Table 3.

**Table 3:** DIF and LD present in the three scales

Alienation dimension	LD/DIF	Items and covariates	χ2	df	*p*
Powerlessness	LD	‘Cannot overcome problems’ & ‘Cannot manage things’	83.54	4	< 0.001
	DIF	‘Feeling helpless’ & ‘Migration status’	14.49	4	< 0.01
		‘Feeling helpless’ & ‘Age group’	16.55	2	< 0.001
		‘Feeling helpless’ & ‘Sex’	21.80	2	< 0.001
Meaninglessness	LD	‘Things have no meaning’ & ‘Not knowing what is going on’	371.61	4	< 0.001
	DIF	‘Things have no meaning’ & ‘Age group’	9.54	2	< 0.01
Social isolation	LD	‘Feeling left out’ & ‘Feeling lonely’	236.71	4	< 0.001
		‘Not feeling close to family’ & ‘No support when unhappy’	127.93	4	< 0.001
		‘Not feeling close to family’ & ‘Not belonging’	39.09	4	< 0.001
	DIF	‘Feeling left out’ & ‘Sex’	26.18	2	< 0.001
		‘Not feeling close to family’ & ‘Age group’	13.49	2	0.001
		‘No support when unhappy’ & ‘Sex’	29.94	2	< 0.001
		‘Feeling lonely’ & ‘Sex’	6.76	2	0.022
		‘Not belonging’ & ‘Sex’	7.65	2	0.022

The analyses of the *powerlessness scale* revealed positive LD between the items ‘Cannot overcome problems’ and ‘Cannot manage things’ meaning that pupils with an indication of alienation in one of the items had higher odds for also having it in the other item. Positive LD suggests that the items are partly redundant. Furthermore, there was DIF of the item ‘Feeling helpless’ in relation to the three covariates: sex, age group and migration status, respectively.

The analyses of the *meaninglessness scale* showed positive LD between the items ‘Things have no meaning’ and ‘Not knowing what is going on’. Besides LD there was DIF of the item ‘Things have no meaning’ in relation to age group.

Finally, the *social isolation* scale contained positive LD between the items ‘Feeling left out’ and ‘Feeling lonely’. The item ‘Not feeling close to family’ and the two items ‘No support when unhappy’ and ‘Not belonging’, respectively, were also locally dependent. The LD between ‘Not feeling close to family’ and ‘Not belonging’ were negative meaning that the degree of indication of alienation decreased in one item when alienation degree increased in the other. There was DIF of four items in relation to the covariate sex. These items were ‘Feeling left out’, ‘No support when unhappy’, ‘Feeling lonely’ and ‘Not belonging’. The DIF of the two last items was only significant at a 5% level of significance (*p* = 0.022) meaning that the evidence of DIF was not as strong as for the other items. It was, however, necessary to include this DIF in order to make the model fit. Last, the analyses showed DIF of the item ‘Not feeling close to family’ in relation to ‘age group.

### Test for global homogeneity and global DIF

Table 4 shows the test results of the analyses for global homogeneity and global DIF using an RM and a GLLRM, respectively.

**Table 4:** Test for global homogeneity and global DIF in relation to covariates by dimension of alienation

			RM			GLLRM		
Alienation dimension	Global homogeneity & DIF	Covariates	χ2	df	*p*	χ2	df	*p*
Powerlessness								
	Global homogeneity		37.7	5	< 0.001	13.1	17	0.728
	Global DIF	Sex	28.4	5	< 0.001	14.3	15	0.506
		Age group	30.6	5	< 0.001	11.0	15	0.756
		SEP	55.3	30	< 0.01	118.9	102	0.121
		Migration status	22.2	10	0.014	31.1	22	0.095
		Physical inactivity	12.0	5	0.035	28.6	17	0.038
Meaninglessness								
	Global homogeneity		160.5	5	< 0.001	10.4	11	0.498
	Global DIF	Sex	11.7	5	0.040	19.2	11	0.058
		Age group	16.9	5	< 0.01	7.2	9	0.616
		SEP	27.5	30	0.599	58.7	66	0.726
		Migration status	10.2	10	0.426	19.3	22	0.627
		Physical inactivity	3.3	5	0.659	11.4	11	0.409
Social isolation								
	Global homogeneity		77.2	9	< 0.001	17.9	31	0.970
	Global DIF	Sex	92.1	9	< 0.001	29.0	15	0.016
		Age group	16.4	9	0.678	30.0	27	0.315
		SEP	54.6	54	0.453	175.3	186	0.702
		Migration status	34.8	18	0.010	89.8	62	0.012
		Physical inactivity	6.6	9	0.678	32.9	31	0.374

The global tests of fit rejected the plain RM (global homogeneity: *p* < 0.001) for all three scales implying that some of the assumptions of the model were compromised. One explanation is LD between items. Furthermore, there was global DIF at a 1% significance level in relation to a varying number of covariates in each of the scales (*powerlessness*: sex, age group and SEP; *meaninglessness*: age group; *social isolation*: age group and migration status). The global tests of fit of the GLLRMs accepted the models. The DIF and LD in the models therefore appeared to correct the flaws of the RMs. This means that no LD and DIF remain besides the LD and DIF taken into account by the final models.

### Item fit

Table 5 shows the items’ overall fit to the model. It shows the observed gamma coefficients for each of the items in the three scales followed by what the expected gamma coefficients would be if they were to fit an RM and GLLRM respectively. The *p*-value shows whether there is a significant difference between the expected and the actually observed gamma coefficient.

**Table 5:** Item fit statistics for RM and GLLRM respectively

		RM			GLLRM		
Alienation dimension & item	Observed gamma coefficients	Expected gamma coefficients	sd	*p*	Expected gamma coefficients	sd	*p*
Powerlessness							
Cannot overcome problems	0.702	0.633	0.024	< 0.001*	0.696	0.021	0.761
Cannot manage things	0.674	0.631	0.024	0.0781	0.694	0.021	0.363
Feeling helpless	0.493	0.633	0.025	< 0.0001*	0.489	0.032	0.903
Meaninglessness							
Things have no meaning	0.570	0.462	0.020	< 0.0001*	0.563	0.018	0.686
Not knowing what is going on	0.570	0.456	0.020	< 0.0001*	0.575	0.018	0.807
School is not preparing me for the future	0.242	0.454	0.021	< 0.0001*	0.240	0.024	0.937
Social isolation							
Feeling left out	0.684	0.547	0.024	< 0.0001*	0.682	0.019	0.922
Not feeling close to family	0.533	0.563	0.027	0.265	0.514	0.029	0.518
No support when unhappy	0.587	0.586	0.029	0.974	0.602	0.029	0.603
Feeling lonely	0.618	0.535	0.022	< 0.001*	0.613	0.020	0.770
Not belonging	0.385	0.550	0.021	< 0.0001*	0.397	0.025	0.625

^*^Significant differences between the expected and actually observed gamma coefficients on a 1% level of significance

Using an RM all three scales contained items where the observed and expected gamma coefficients differed significantly on a 1% significance level; two items in the powerlessness scale and three items in each of the scales for meaninglessness and social isolation respectively, had significantly differing values indicating a poor fit of these items. When using GLLRM the *p*-values increased markedly and became clearly insignificant for all items in the three scales indicating a good fit to the GLLRM.

The three scales’ test information functions are presented in Additional files 1, 2 and 3: Figure S1, S2 and S3 stratified by the respective DIF groups of the scales (powerlessness: sex, age group and migration status; meaninglessness: age group; social isolation; sex and age group). They illustrate at which level of the latent variable (theta) the test information is maximal and thereby, also where measurement precision is highest [49]. The test information functions of the powerlessness scale (Additional file 1: Figure S1) and meaninglessness scale (Additional file 2: Figure S2) were very similar regardless of the respective DIF group. For social isolation the maximal test information was slightly higher for boys and slightly shifted toward the lower end of the scale compared to girls,

The reliabilities of the three scales measured as test-retest were relatively low ranging between 0.35–0.69 (powerlessness: 0.40–0.69, meaninglessness: 0.35–0.36, and social isolation: 0.52–0.61). Nevertheless, as the scales fitted an RM when using GLLRMs they can be regarded as essentially valid, essentially objective and sufficient as shown in Kreiner and Christensen [28, 33].

## Discussion

In this study, we developed scales for measurement of three of the alienation variants in Seeman’s conceptualization [10, 11]: powerlessness, meaninglessness and social isolation. These alienation variants are particularly relevant for adolescent health research as the feeling of empowerment and having supportive social relations, are important aspects of a positive youth development [2, 3, 6]. The three scales appear to be face and content valid based on the conceptual, qualitative and psychometric work conducted during the development of the scales. All three scales contained some LD and DIF, however, only to a limited degree, which could be taken into account using GLLRMs. Adjusted for DIF and LD the three scales can be described as being essentially construct valid, and essentially objective with reference to Kreiner and Christensen’s definitions [28, 33] in this sample of 13 and 15-year-olds. As each of the three scales fitted an RM when using GLLRMs they all fulfil the psychometric properties of a good reflective scale.

Like previous alienation studies [20, 22–26], our study suggests that it is possible to develop valid scales for the measurement of alienation among adolescents. The three scales developed in this study share their use of Seeman’s conceptualization of alienation with previous scales. Two of these previous studies likewise used HBSC data using Danish and Portuguese data, respectively [20, 26]. Previously, we constructed a simple index of overall alienation representing aspects of social isolation, powerlessness and cultural estrangement [20]. Inspired by this study Tomé et al. [26] developed scales for social isolation, normlessness, powerlessness and meaninglessness, using the Portuguese HBSC survey. Our study is in concordance with these earlier studies in suggesting that alienation is a relevant concept in adolescent research and can be measured based on data from the HBSC survey. Our study furthermore suggests that such alienation scales may meet the most demanding criteria for construct validity: unidimensionality, monotonicity, local independence between items, and no DIF.

Several scholars have focused on issues of reliability and validity in the measurement of alienation. Dean [22] contributed with a thorough categorization of items into three scales and addressed internal consistency and convergent validity, however, not in an adolescent setting. Jessor and Jessor [23] contributed with a scale measuring adolescent alienation. Several studies have investigated the dimensionality of the concept among adolescents [15, 24–27, 50]; some of them also developed scales measuring adolescent alienation [24–27]. Some studies [20, 24, 26, 27] furthermore investigated the construct validity by comparing the pattern of group difference to the pattern expected from theory [24], by confirmatory factor analysis [26, 27] or by analyses for DIF [20]. Modern item response theory does, nevertheless, provide a more coherent approach to the study of construct validity using RM and GLLRM.

Seeman [10] emphasized that even though some of his alienation variants can be related, no theoretical structure exists. The multidimensionality of alienation has been confirmed but there are studies indicating that some variants are more loosely related to alienation as a general concept than others [50] and that some variants (normlessness and meaninglessness) are more closely related to each other than other variants [50, 51]. Therefore, one study [50] concluded that using only one general term of alienation ‘may hamper our understanding of the specific nature and development of alienation during childhood and adolescence’. This is coherent with Seeman’s conceptualization [12]. Together with the psychometric demand of unidimensionality this suggests that future alienation scales should continue to treat the measure alienation by distinct scale for the alienation variants.

The present study focuses on three specific variants of alienation: powerlessness, meaninglessness and social isolation. These three variants were chosen because they are specifically relevant for adolescent psychosocial development. The choice was also based on considerations that some of Seeman’s variants may be relatively abstract to 13- 15-year-olds and taking into account that the scales should be applicable in a school survey among adolescents. One example is the alienation variant self-estrangement, which indicates that some ideal human condition exists [9]. Self-estrangement is seldom addressed in research. According to Williams and Cullingford [9], this is most likely because it is less accessible via testing, particularly among children and adolescents.

One way to ensure content validity is to conduct cognitive interviews among respondents early in the process in order to identify the most relevant content of a scale and to ensure that the content is appropriate for the age group. Additionally, such interviews may also help to enhance the phrasing of the items. We did not use cognitive interviews in the early phase of item development in this study and propose to apply this technique in future scale development studies. The development of the items was, based on a thoroughly conducted conceptual work, previous items on alienation or related concepts, discussions with researchers with expertise on adolescent psychosocial health and six focus group interviews comprising 44 13- and 15-year-olds. This process suggests that the content validity is acceptable among 13 to 15-year-olds. Also, the tests for DIF in relation to age group revealed only a limited amount of DIF, which can be adjusted for using GLLRM. Still, the content validity of the scales may be enhanced by using cognitive interviews in future development of the scales. Adolescence is a time of rapid development and the present study’s results apply to the specific frame of reference the scales have been tested in (e.g. similar age group only). The three scales’ test information functions were very similar for 13-year olds and 15-year olds. Still, it is considered necessary to re-address the validity of the scales before using the scales in substantially older samples. Based on qualitative focus group interviews among 11-year-olds the scales should not be used in a younger sample as these interviews indicated the content or phrasing of the items was either irrelevant or difficult to understand for this age group.

The three scales are relatively short compared to the scales developed in previous studies. This makes the scales easily manageable in surveys but may also have affected the reliability. The reliability of each of the three scales, measured as test-retest, was low ranging between 0.34 and 0.69. A low reliability can be considered a limitation, but is mostly a concern in small samples where the risk of type 2 errors is inflated. This means that the three scales would serve as a poor diagnostic tool for identifying single alienated adolescents. When applied in research the low reliability can be considered of less importance for two reasons. First, because the reliability increases by sample size; second, because the test-true score reliability which provides a better measure of the performance of the measurement instrument, is higher. Expressed by test-true score correlation, the reliabilities of the scales ranged between 0.62–0.83 (powerlessness: 0.64–0.83, meaninglessness: 0.62–0.65, social isolation: 0.73–0.77).

We used trichotomized response categories to create a conceptual symmetry. Thus, it appears natural to investigate how the scales would work with rephrased three-option response scales in a future validity study in order to re-test the criterion-related construct validity. A three-option version would, however, pose the risk that the respondent does not feel there are enough or sufficiently nuanced response options to provide an adequate answer. It may therefore be preferable with five response options and subsequently collapse the categories.

The prevalence of alienation varies considerably by item within all three scales. This is appropriate because the scales are then able to identify both mild and severe levels of alienation. Another merit of the scales is that information bias was sought reduced by several means: First by addressing face validity through pilot testing of the items and second, by a high construct validity confirmed by the GLLRMs analyses. Finally, the anonymity of the pupils encouraged honest answers. Regarding selection bias the response rates were high. Still, it is possible that the most alienated pupils were absent in a higher degree than non- or low-alienated students. Unfortunately, the anonymity made an analysis of the non-respondents impossible. As the conditional distribution of item responses given the total score does not depend on the distribution of the latent variables in RMs, the analysis of scale validity and essential objectivity as defined by Kreiner and Christensen [28, 33] would, not be violated if the non-respondents were more alienated than the respondents were.

The psychometric properties, content and face validity of the three scales suggest that the scales developed in this study can be used in large-scale surveys. One next step is to test the three new scales of alienation in research examining the relation between alienation and outcomes on adolescent health, behavior and wellbeing. The current scales should not be used for screening purposes (e.g. identifying single adolescents at risk) due to the relatively low reliabilities. Another important step in future work is to investigate if items could be added to the scales and thereby increase reliability. Our study suggests that such work should be founded in Seeman’s conceptualization, include thoroughly conducted qualitative interviews and examination of the construct validity.

## Conclusion

The three alienation scales on powerlessness, meaninglessness and social isolation appear to be face and content valid. All three scales fulfill the psychometric properties of a good reflective scale. This suggests that the scales may be appropriate in future studies to examine the relation between alienation and a range of adolescent health outcomes such as health, behavior and wellbeing. Because alienation refers to specific processes in the transactions between the individual and the social environment the scales may have the potential to offer more specific explanation of the processes behind these associations. For screening purposes, the scales still need further development.

## Additional files

Additional file 1: **Figure S1.** Test information function: Powerlessness stratified by sex, age group and migration status. (DOCX 108 kb)

Additional file 2: **Figure S2.** Test information function: Meaninglessness stratified by age group. (DOCX 31 kb)

Additional file 3: **Figure S3.** Test information function: Social isolation stratified by sex and age group. (DOCX 51 kb)

## Abbreviations

CLR: Conditional likelihood ratio
DIF: Differential item functioning
GLLRM: Graphical loglinear Rasch model
HBSC: Health Behaviour in School-aged Children
IRT: Item response theory
LD: Local dependency
MVPA: Moderate to vigorous physical activity
OSC: Occupational social class
RM: Rasch model
SEP: Socioeconomic position
VPA: Vigorous physical activity
