# Supplementary material for: Measurement of alienation among adolescents: construct validity of three scales on powerlessness, meaninglessness and social isolation
Source: J Patient Rep Outcomes. 2018 Mar 16;2:14. doi: 10.1186/s41687-018-0040-y (PMC5934919; doi:10.1186/s41687-018-0040-y)
Supplement: Supplementary file 1 — Figure S1. Test information function: Powerlessness stratified by sex, age group and migration status. (DOCX 108 kb) [file 41687_2018_40_MOESM1_ESM.docx]

**Suppl. Figure 1. Test information function: Powerlessness stratified by sex, age group and migration status**

| **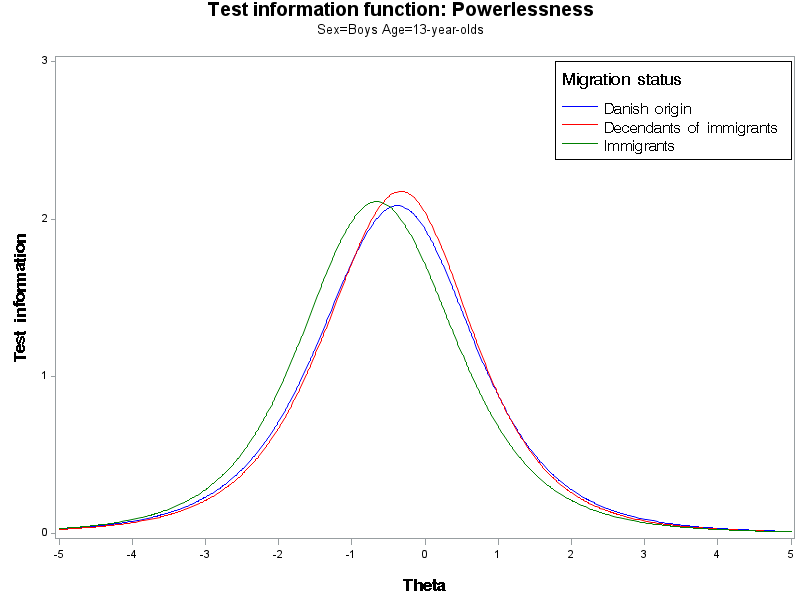**  **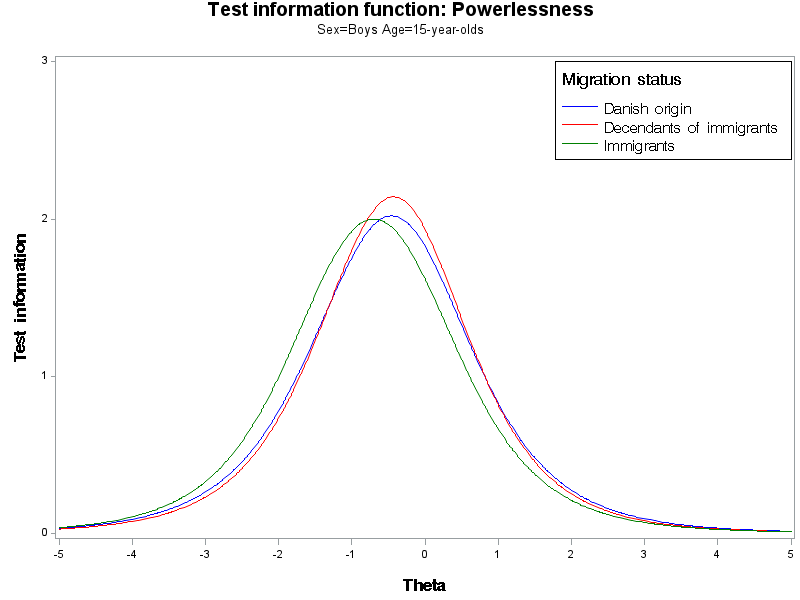** |
| --- |

**Suppl. Figure 1 continued. Test information function: Powerlessness stratified by sex, age group and migration status**

| **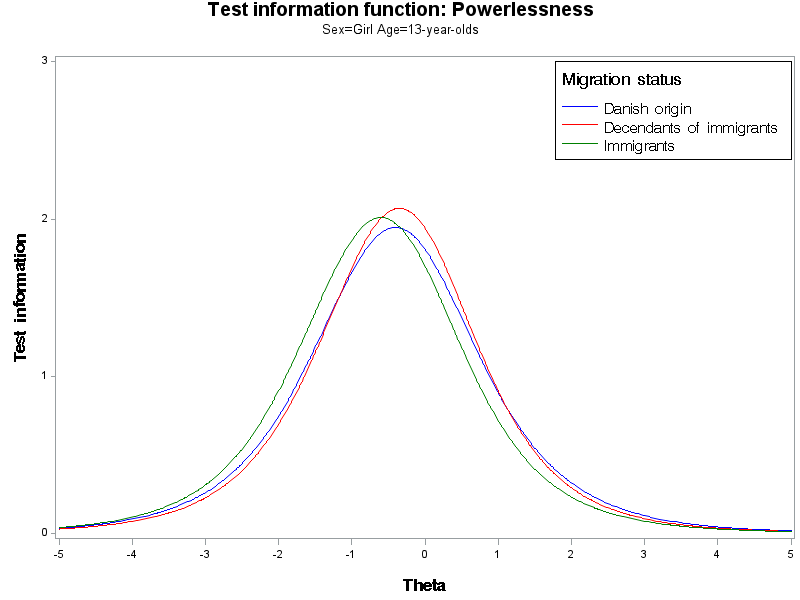**  **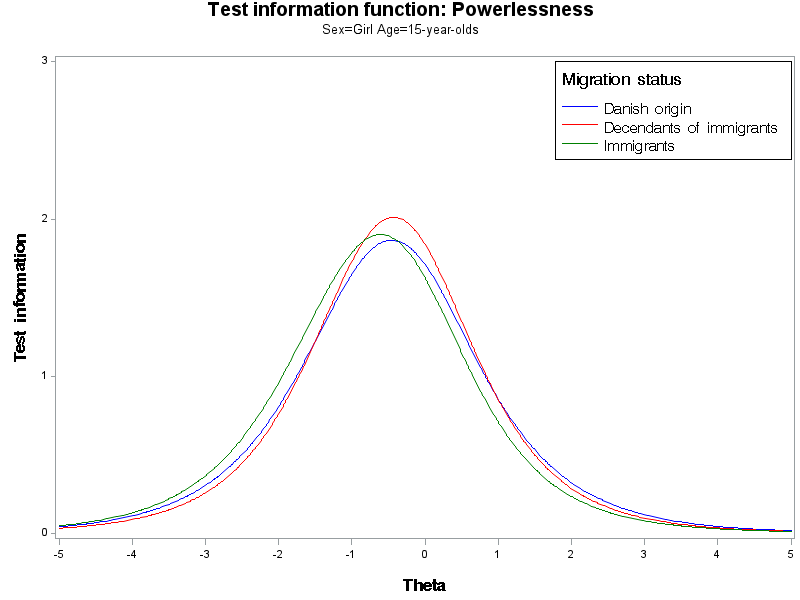** |
| --- |
