# Supplementary figures and images for: Measurement of alienation among adolescents: construct validity of three scales on powerlessness, meaninglessness and social isolation
Source: J Patient Rep Outcomes. 2018 Mar 16;2:14. doi: 10.1186/s41687-018-0040-y (PMC5934919; doi:10.1186/s41687-018-0040-y)

**Suppl. Figure 2. Test information function: Meaninglessness stratified by age group**

| **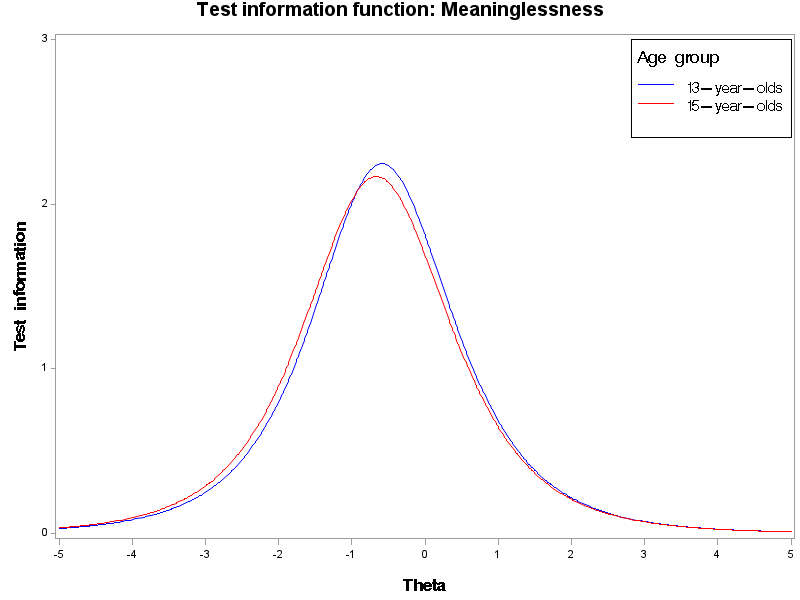** |
| --- |

Supplement: Supplementary file 2 — Figure S2. Test information function: Meaninglessness stratified by age group. (DOCX 31 kb) [file 41687_2018_40_MOESM2_ESM.docx]

**Suppl. Figure 3. Test information function: Social isolation stratified by sex and age group**

| **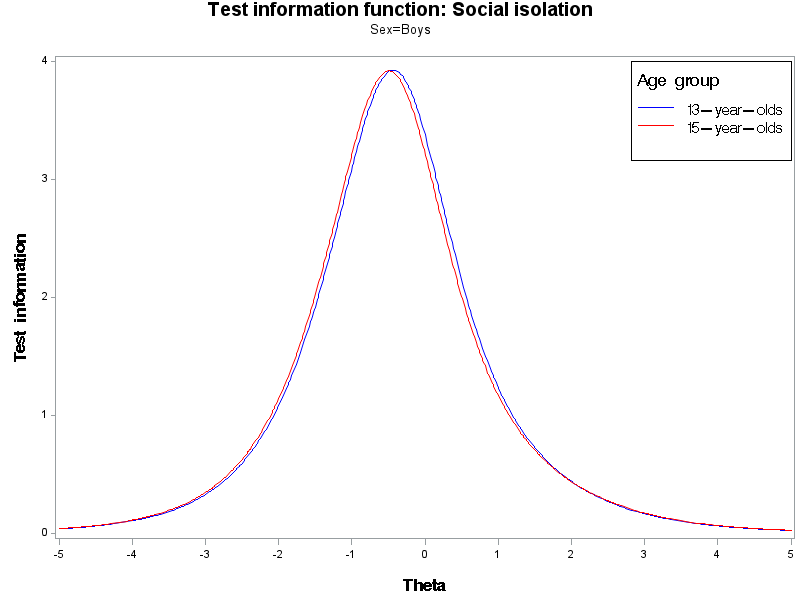**  **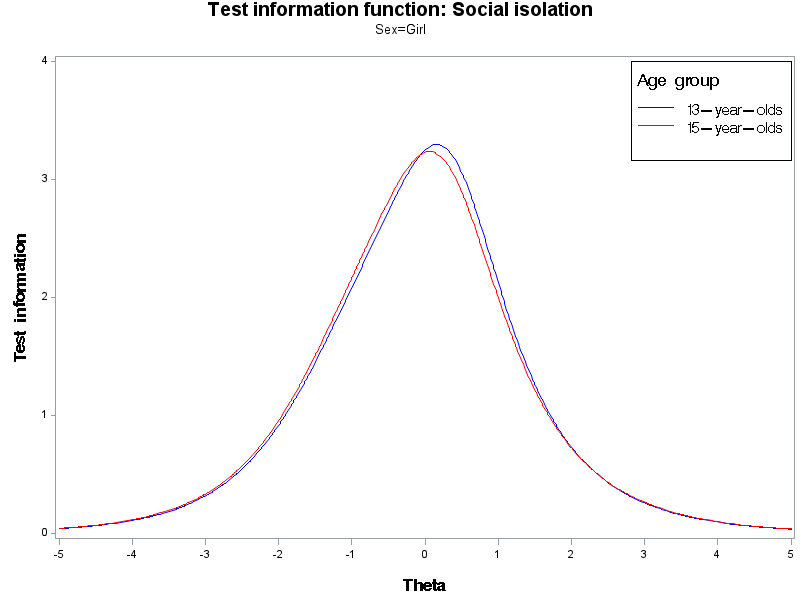** |
| --- |

Supplement: Supplementary file 3 — Figure S3. Test information function: Social isolation stratified by sex and age group. (DOCX 51 kb) [file 41687_2018_40_MOESM3_ESM.docx]
